# The ESCRT machinery: new roles at new holes

**DOI:** 10.1016/j.ceb.2015.12.001

**Published:** 2016-02

**Authors:** Y Olmos, JG Carlton

**Affiliations:** Division of Cancer Studies, Section of Cell Biology and Imaging, King's College London, London SE1 1UL, United Kingdom

## Abstract

The ESCRT machinery drives a diverse collection of membrane remodeling events, including multivesicular body biogenesis, release of enveloped retroviruses and both reformation of the nuclear envelope and cytokinetic abscission during mitotic exit. These events share the requirement for a topologically equivalent membrane remodeling for their completion and the cells deployment of the ESCRT machinery in these different contexts highlights its functionality as a transposable membrane-fission machinery. Here, we will examine recent data describing ESCRT-III dependent membrane remodeling and explore new roles for the ESCRT-III complex at the nuclear envelope.

**Current Opinion in Cell Biology** 2016, **38**:1–11

This review comes from a themed issue on **Cell architecture**

Edited by **Matthieu Piel** and **Margaret Gardel**

For a complete overview see the Issue and the Editorial

Available online 15th January 2016

**http://dx.doi.org/10.1016/j.ceb.2015.12.001**

0955-0674/© 2016 The Authors. Published by Elsevier Ltd. This is an open access article under the CC BY license (http://creativecommons.org/licenses/by/4.0/).

## Introduction

**Figure 1:**
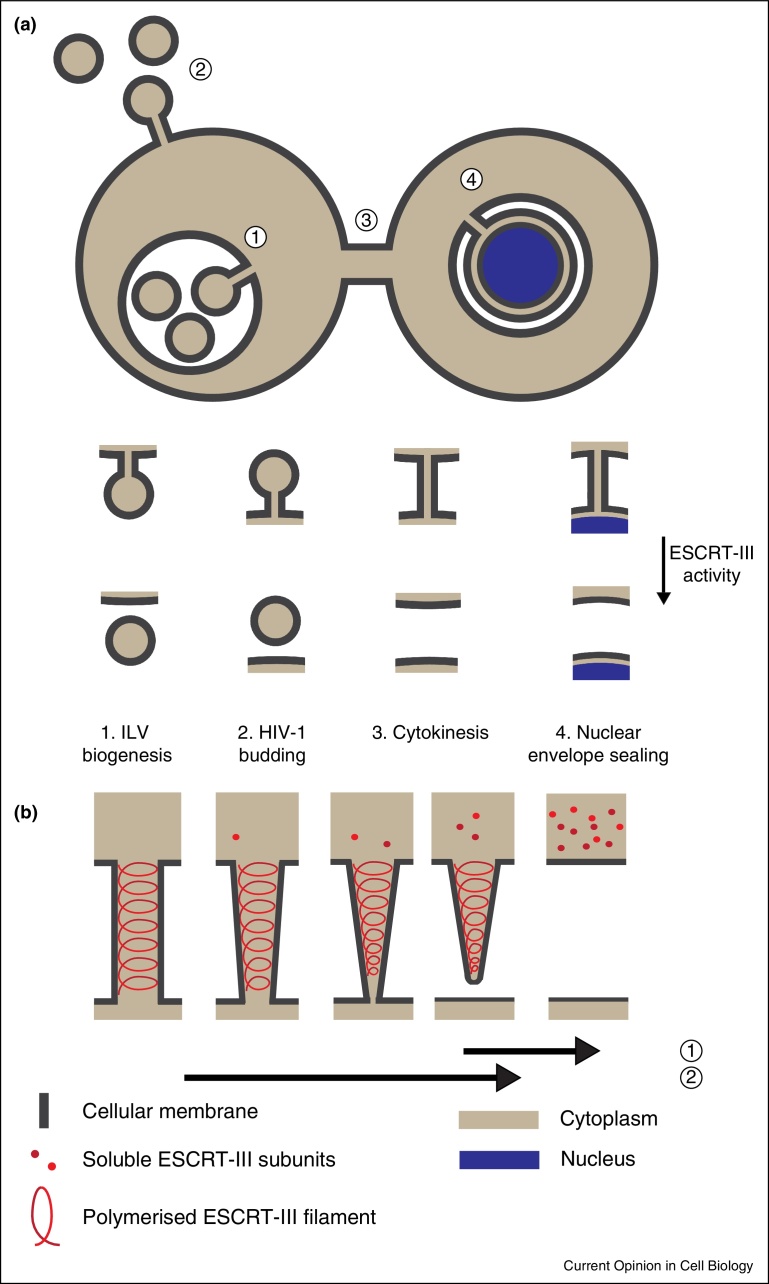
Sites and models of ESCRT-III activity. **(a)** Cartoon depicting sites of topologically-equivalent membrane remodeling performed by the ESCRT-III complex. In all cases, ESCRT-III provides an activity allowing resolution of the membranous stalk with the concomitant separation of the two membranes previously connected by the stalk. This separation achieves release of ILVs (1), enveloped viruses such as HIV-1 (2), daughter cells (3) and separation of previously connected inner and outer nuclear membranes (4). Bottom schematic depicts membrane separation in each case achieved through ESCRT-III activity. **(b)** Models for ESCRT-III driven membrane fission. Filaments of polymerised ESCRT-III subunits are thought to assemble inside a membranous stalk, connecting two parental membranes. ESCRT-III assembly, either via the shape of the formed holo-polymer (dome model), or through constriction of the ESCRT-III filament (purse string model), narrows the stalk. This narrowing presumably makes it energetically more favourable to separate the membranes, rather than persist with membranes connected by a highly-curved, thin, membranous stalk. Models propose that the AAA-ATPase VPS4 acts either to tighten the filament, through sequential extraction of polymerised CHMPs (purse string model, VPS4 activity needed throughout remodeling event (1)) or through the induction of conformational changes in the CHMPs by direct interaction. Alternatively, membrane fission is accomplished through formation of the ESCRT-III holo-polymer (dome model, with VPS4 acting after fission to disassemble ESCRT-III filaments for subsequent rounds of assembly (2)). Period of VPS4 activity indicated by arrow.

The Endosomal Sorting Complex Required for Transport (ESCRT) machinery is an evolutionarily-conserved, multi-subunit membrane remodeling complex (Table 1). Originally identified in yeast for its essential role in the biogenesis of intraluminal vesicles (ILVs) upon a class of endosome called the multivesicular body (MVB) [1, 2, 3], its roles in mammalian cells have been expanded to encompass a number of topologically equivalent membrane remodeling events. ILV biogenesis requires the endosome's limiting membrane to be pushed away from the cytosol to generate a nascent bud connected via a membranous stalk to the endosomal limiting membrane. Subsequent severing of this nascent ILV from the limiting membrane requires a membrane fission activity that acts on the cytosolic face of this membranous stalk (Figure 1a). It is thought that the ESCRT-III complex provides this activity, whilst upstream ESCRT-components (ESCRT-I and ESCRT-II) coordinate this fission with the forming and sorting of ubiquitinated cargo onto the nascent ILV [4•, 5•]. As well as being a prerequisite to lysosomal degradation, the contents of MVBs can be released upon fusion of the MVB with the plasma membrane — the released ILVs are exosomes and as their formation is ESCRT-dependent, ESCRTs thus offer a layer of control over this route of intercellular communication [6, 7]. Whilst an endosomal function for ESCRTs has been demonstrated across a range of organisms, the topologically unique membrane remodeling that ESCRT-III complex can perform has allowed this machinery to be co-opted by a number of cellular factors and to drive a diverse range of physiological and pathophysiological cellular processes across organisms from *Archaea* to animals [8].

In mammalian cells, ESCRT-components have been implicated as host-factors for the release of enveloped retroviruses such as HIV-1 or Ebola [9•, 10•, 11•, 12•]. The structural proteins of these enveloped viruses encode peptide-ligands (Late, or L-domains), that are able to directly bind ESCRT-proteins. Here, the ESCRT-III complex, recruited via TSG101 and ESCRT-I or by the ESCRT-III accessory protein ALIX [13, 14], functions to sever the membranous stalks connecting budded virions with the plasma membrane in a manner topologically analogous to the membrane fission reaction performed to release ILVs at the MVB (Figure 1a). Whist largely implicated in the budding of virions from the plasma membrane, ESCRT proteins have also been involved in the lifecycle and release of viruses such as Epstein-Barr virus that traverse intracellular membranes such as the nuclear envelope [15] and Herpes Simplex viruses [16] that buds through the nuclear envelope and again into the lumen of membranes organelles during secondary envelopment, suggesting that ESCRT-III may again be recruited for the membrane remodeling allowing these envelopment events.

In addition to a pathophysiological role in viral replication, the ESCRT proteins TSG101 and ALIX are recruited to the midbody of dividing cells through interaction with the midbody protein CEP55 [17•, 18•, 19]. Here, they function to recruit the ESCRT-III complex to perform a topologically equivalent membrane fission during cytokinetic abscission (Figure 1a), allowing the separation of daughter cells and the completion of cell division [20, 21]. ESCRT-III proteins have additionally been implicated in the repair of damaged regions of the plasma membrane, where they are recruited in a Ca^++^-dependent manner by ALG2 [22•, 23•] to sever protruding, damaged regions of the plasma membrane in a manner similar to that employed in viral release. Further, ESCRT-III has been shown to play roles in autophagy [24, 25], neuronal pruning [26], material transfer at the immunological synapse [27] and release of exosomes [6]. This article will not focus on these classical (or even those more esoteric) functions of the ESCRT-machinery; readers are referred to myriad excellent reviews on this subject elsewhere [28, 29, 30, 31]. Rather, we will focus on newly published data describing new roles for ESCRT-III during cell division.

## ESCRT-III

**Figure 2:**
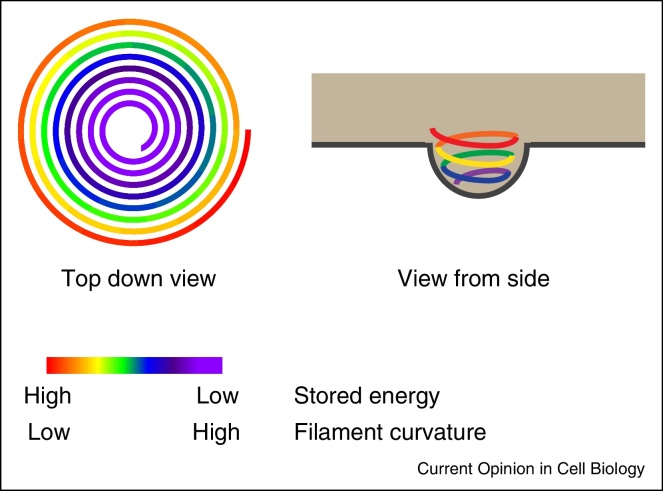
Energetic considerations in ESCRT-III assembly. Cartoon depicting the energy stored in helical polymers of ESCRT-III subunits and the suggestion that this energy is released to allow membrane deformation.

**Figure 3:**
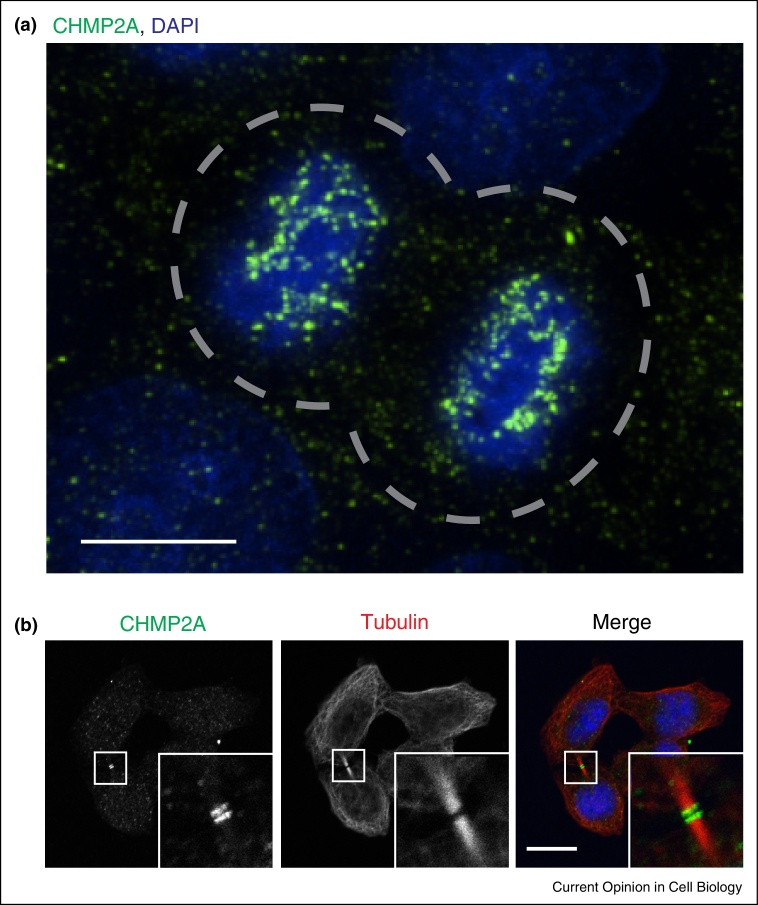
Mitotic ESCRT-III localization. Immunofluorescence analysis of HeLa cells stained with antibodies against endogenous CHMP2A or tubulin as indicated, demonstrating CHMP2A localization to the reforming nuclear envelope **(a)** or the midbody during cytokinesis **(b)**. For methods and more detailed images, see Olmos *et al.* (2015). In A, dotted outline depicts cell border, images obtained by deconvolution of widefield images (a) or confocal imaging (b). Scale bar is 10 μm.

ESCRT-III is the hypothesised membrane fission complex within the ESCRT-machinery and is directed to its sites of action through interaction with upstream ESCRT-components, themselves localized by various adaptor proteins as described above. ESCRT-III subunits (CHMPs, for *Ch*arged *M*ultivesicular Body *P*roteins [32], or *Ch*romatin *M*odifying *P*roteins [33]) transition between soluble and polymerising states, and assemble in a defined order to form a membrane-remodeling filament that brings about membrane fission. CHMP proteins have long been proposed to exist in closed and open conformations, with the hypothesis that the open conformation represented a filament-forming state. Recent cryo-EM data has at last resolved the molecular structure of a CHMP (CHMP1B) in the ‘open’ conformation and revealed it to adopt an extended α-helical V-shaped structure that bound to proximal open-forms of itself in a regular array to form a filament in a co-polymer with the N-terminal domain of IST1 [34^••^]. Interestingly, IST1 in this filament was found on the outside of this filament in the closed conformation, whereas CHMP1B in this filament was found in the open conformation. Of note, CHMP1B and IST1 play atypical roles in ESCRT-biology, being dispensable for HIV-1 release and degradative cargo sorting and whilst IST1 functions in cytokinesis [35, 36] and at the nuclear envelope [37^••^] (see below), IST1 is also necessary for scission of recycling carriers leaving endosomes [38]. Consistent with this, the CHMP1B:IST1 filament contained a luminal (rather than peripheral) membrane-binding surface, suggesting a function as a scaffold around membranous stalks, rather than inside them [34^••^]. Indeed, the IST1:CHMP1B filaments were straight and not spiral indicating that they may be better suited to constriction of a tubule rather than the tightening of a stalk (as described below). These data indicate that a heteropolymeric ESCRT-III filament can accommodate CHMPs in different activation states and suggest that the open form represents the membrane-binding conformation. The structure of an ‘open’ CHMP will hopefully guide our understanding of how filaments formed from CHMP-2, CHMP-4 and CHMP-3 generate and stabilise the negative curvature required for ESCRT-III dependent membrane separation. Mechanisms of ESCRT-III assembly and disassembly have been reviewed well elsewhere [29, 31, 39] and models of how ESCRT-III achieves the terminal membrane fission have been proposed [39, 40, 41]. These models of ESCRT-III function remain largely untested, but are linked by the basic premise that ESCRT-III assembles as a spiral filament on the cytosolic face of a membranous stalk, and provides an activity allowing resolution of this stalk and separation of the two previously connected membranes (Figure 1b). Exactly how this scission occurs is not known, but remodeling of the ESCRT-III polymer may contribute either to membrane deformation, or to the membrane separation. It is known that ESCRT-III proteins form spiral filaments in vitro, suggesting that the oligomer may have a preferred curvature. It was recently shown that filaments formed from Vps32 (the *C. elegans* homologue of CHMP4B/Snf7) could accommodate a range of bending angles [42^••^] and analysis of Snf7 filament assembly on supported bilayers have added some new considerations into how filament assembly may drive membrane remodelling. Using a combination of high-speed atomic force microscopy and electron microscopy, circular arrays of Snf7 with a preferred diameter of 25 nm were observed to break and seed polymerisation of helical Snf7 filaments that grew radially on supported lipid membranes [43^••^]. As these spirals grew, the degree of filament curvature was necessarily reduced to accommodate the growing spiral, putting elastic strain on the filament. The growing Snf7 filament was suggested to act as a spiral-spring, storing elastic energy as the filament extended; indeed, molecular dynamics simulations predict the free energy state of Vps32 filaments is dramatically increased at the edge of the spiral [42^••^] and measurements of membrane tension [43^••^] were increased upon Snf7 polymerisation. Upon reaching confluency, spiral growth was inhibited by lateral interactions, and spirals released their stored energy through deformation, suggesting that in a cellular environment, this release of elastic energy could be transferred to the membrane. The authors propose that this energy drives ESCRT-III-dependent membrane deformation (Figure 2), perhaps by moving leaflets of the bilayer relative to each other. Whilst this is a likely scenario, it should also be noted that ESCRT-III functions in a number of situations where membrane deformation is dispensible (for example, during virion release, nuclear envelope sealing and cytokinetic abscission; here the membranous stalk to be resolved is pre-existing) and it is possible that the energy stored in these filaments is alternatively used to drive membrane separation rather than membrane deformation. An additional point that these data raise is a consideration of scale. Whilst viral stalks, ILV stalks, and stalks to be resolved in the nuclear envelope are of similar dimensions (30–50 nm), the midbody is much larger, with ESCRT-III components being observed when the midbody has diameters of approximately 1 μm (Figure 3b [44]). This will necessitate the formation of a filament with a very low curvature, that would be predicted to store great deal of elastic energy, perhaps (given the larger diameter of ESCRT-III-dependent cytokinetic filaments [44]) necessitating partner proteins to prevent breakage, and be capable of transferring this greater energy to the membrane which may be necessary for efficient constriction and abscission of this larger structure.

In addition to individual ESCRT-III subunits, the MIT-domain containing AAA-ATPase, VPS4, is recruited to short peptide motifs in the C-terminus of ESCRT-III subunits to allow for ESCRT-III filament disassembly and subunit reuse in subsequent rounds of ESCRT-activity — VPS4 binding could also remodel and constrict the Vps32 spirals in an ATPase-independent manner [42^••^], suggesting that it too could modulate the elastic energy within the ESCRT-III filament. Interestingly, ESCRT-III subunits are additionally capable of recruiting alternate AAA-ATPases, such as the microtubule-severing enzyme Spastin [45], which have important roles in coordinating ESCRT-III-dependent membrane and cytoskeletal modeling as described below.

## ESCRTs on the nuclear envelope — NPC surveillance

The Nuclear Pore Complex (NPC) is an ancient, conserved and long-lived structure that allows gated exchange between nucleoplasm and cytoplasm, allowing the establishment of proper nucleo-cytoplasmic compartmentalization [46]. A recent epistasis screen uncovered an unexpected role for the core ESCRT-III complex in extracting defective NPCs, thus ascribing a surveillance role for this complex at the nuclear envelope [47^••^]. In this role, ESCRT-III was recruited to the nuclear envelope through interaction of Snf7 with the *L*ap2-*E*merin-*M*AN1 (LEM) family proteins Heh1 and Heh2 that had previously been implicated in NPC quality control [48]. In yeast lacking ESCRT-III components, Vps4 or Heh2, aberrantly assembled NPC components were sequestered within a domain on the nuclear envelope termed the SINC (*S*torage of *I*mproperly assembled *N*P*C*s). Unlike mammalian cells who disassemble and reassemble their nuclear envelope during each open mitosis, yeast undergo a closed mitosis and thus require surveillance mechanisms such as ESCRT-III (and parallel quality-control mechanisms such as the ASI-complex may tie the ERAD pathway into surveillance of other ubiquitinated proteins at this organelle [49, 50]), to extract malfunctioning NPC components. Whether a similar surveillance role for ESCRT-III exists in higher organisms remains to be established, but is likely to be complicated by the open-mitosis that occur in these systems. The basis for ESCRT-III dependent extraction of defective Nups likely involves ubiquitin as proteosomal (but not vacuolar) inhibition impaired this extraction [47^••^], however, the exact mechanism by which ESCRT-III extracts membrane proteins and directs them to the proteasome (rather than the lysosome) is unknown. Whilst the ESCRT-machinery commonly processes ubiquitinated proteins, the absence of ubiquitin-recognition domains within ESCRT-III suggests that additional components of this extraction machinery that direct ubiquitinated nucleoporins for ESCRT-III mediated extraction may exist. Webster and Lusk have proposed attractive models for the operation of ESCRT-III in this context [51] — given the role of AAA-ATPases such as VPS4 in disassembling and solubilizing protein complexes, it is possible that recruited Vps4 plays a direct role in the extraction of these defective intermediates, or uses the physical link between ESCRT-III bound nucleoporins to extract them indirectly, via extraction of the ESCRT-III subunit. Alternatively, budding profiles at the INM and intra-membrane vesicular structures were observed in δ*vps4* cells suggesting that these defective nucleoporins may be cleared through vesicular-budding into the intermembrane space [47^••^]. Whilst this model is topologically satisfying from an ESCRT-III perspective, it is unclear how vesicles in the inter-membrane space could then access the proteasome for degradation.

## ESCRT-III in nuclear envelope reformation

Cell division in eukaryotes involves extensive remodeling of the nuclear envelope (NE) to ensure proper segregation of nuclear and cytoplasmic contents. Higher eukaryotic cells divide by open mitosis, in which the nuclear envelope is broken down at mitotic onset to allow the chromosomes to access the mitotic spindle. This process involves the disassembly and dispersal of all the main elements of the nuclear envelope, including the nuclear membranes, NPCs and the lamina. Upon mitotic onset, some ESCRT-III subunits are subject to phosphorylation, which may render them inactive until they are dephosphorylated during mitotic exit [52]. After chromosome segregation, during telophase, a new nuclear envelope is reformed around each daughter nuclei in order to re-establish nucleo-cytoplasmic partitioning [53, 54, 55, 56]. Nuclear envelope reformation is a two-step process that requires extensive membrane fusion. First, ER membranes, proposed to be either cisternal [57, 58] or tubular [59, 60], attach to chromosomes and spread out, coating the chromatin surface in double-membrane sheets. Secondly, and in order to form a completely sealed nuclear envelope, it is necessary to close any remaining holes or gaps within the double nuclear membranes through the process of annular fusion [55]. In vitro studies of nuclear envelope reassembly in *Xenopus* egg extracts showed that both steps of nuclear envelope reformation are regulated by the p97 AAA-ATPase in association with its adaptor proteins p47, in the case of membrane delivery and nuclear expansion, or UFD1/NPL4 for the annular fusion event [61^•^]. The mechanism by which annular fusion occurs has remained largely unknown, but the topology of this process is identical to cytokinetic abscission as the closure of holes in the re-forming nuclear envelope requires a fission event to separate the connected INM and ONM (Figure 1a). Whilst localization of ESCRT-III subunits to the telophase nuclei was previously reported [33], recent studies [37••, 62••] have added mechanistic insights into this process by demonstrating that ESCRT-III plays an essential role in the sealing of the nuclear envelope during late stages of cell division. High-resolution microscopical approaches, including use of Structured-Illumination-Microscopy (SIM) and correlative light and electron microscopy (CLEM), demonstrated the transient recruitment of ESCRT-III components and VPS4 to sites of annular fusion in the reforming nuclear envelope (Figure 3a) [37••, 62••]. Importantly, these sites of annular fusion comprised membranous stalks connecting INM and ONM and were of similar dimensions to the stalks connecting nascent virions to the plasma membrane or intraluminal vesicles to the endosomal limiting membrane (30–50 nm) [62^••^]. The transient nature of this recruitment was exposed through live imaging approaches, demonstrating recruitment times of between 1 and 5 min, paralleling well the timings of ESCRT-III recruitment to sites of HIV-1 release at the plasma membrane [63, 64, 65]. ESCRT-III assembly on the reforming nuclear envelope occurs through a canonical pathway, with sequential recruitment of CHMP4, CHMP3 and CHMP2 subunits [62^••^], highlighting its similarities to other ESCRT-III-driven processes. Furthermore, the previously uncharacterized CHMP7 protein was identified as an essential recruiter of CHMP4B during nuclear envelope reformation, giving at last a role for this protein [37^••^]. A yeast CHMP7, long thought erroneously to not exist, has recently been implicated in NPC assembly [66] which may further link NPC surveillance and membrane sealing functions at this membrane. CHMP7 is unique amongst ESCRT-III subunits in that it contains an extended N-terminal domain of unknown function — whether this domain specifies a role in nuclear envelope functionality remains to be established.

Importantly, correlative electron tomography analysis demonstrated that ESCRT-III depletion results in the persistence of unsealed holes in the post-mitotic nuclear envelope. As shown in compartmentalization assays, these unsealed nuclear envelopes are functionally leaky, failing to ensure proper nucleo-cytoplasmic partitioning of a variety of import reporters and ultimately leading to the appearance of DNA damage foci at presumptive unsealed sites, highlighting the important role ESCRT-III has in protecting the genome from damage during mitotic exit [37••, 62••]. An interaction was also described between the p97 adaptor protein UFD1 and the late-acting ESCRT-III subunit CHMP2A. p97/UFD1 has a previously described role in nuclear envelope sealing [61] and regulates extraction of ubiquitinated Aurora-B from chromatin, allowing chromatin decondensation as the envelope is closed [67, 68]. Although CHMP2A is recruited to the forming nuclear envelope through interaction with CHMP4 proteins, UFD1 depletion also impaired recruitment of CHMP2A to this membrane — in this context, UFD1's ability to interact with the autoinhibitory helix of CHMP2A suggests that this complex may play an additional role in regulating CHMP2A incorporation and assembly of ESCRT-III at the reforming nuclear envelope.

**Figure 4:**
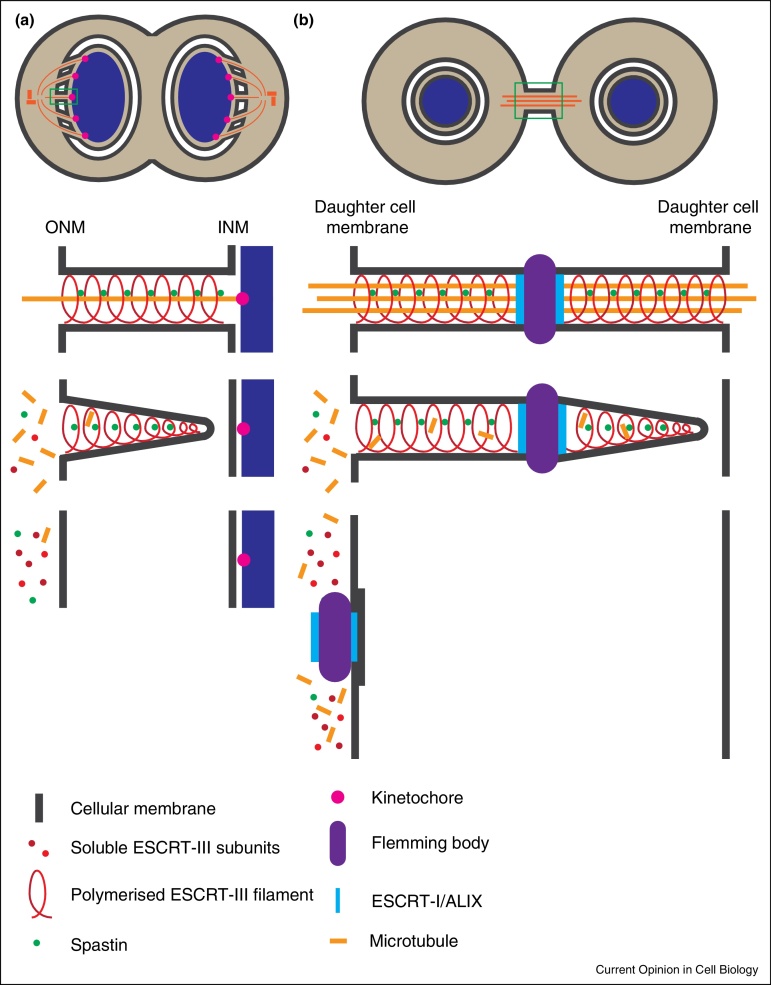
Mitotic co-ordination of membrane and cytoskeletal remodeling by ESCRT-III. A. Cartoon depicting similarities between ESCRT-III dependent nuclear envelope reformation **(a)** and cytokinesis **(b)**. Enlargement of green-boxed region given in lower cartoon. In both cases, prior to ESCRT-III-dependent membrane resolution, microtubules must be coordinately removed through the action of the AAA-ATPase Spastin, which is recruited to the site of remodeling through interaction with IST1 during nuclear envelope reformation, or IST1/CHMP1B during cytokinesis. In the case of cytokinesis, abscission occurs at a constriction upon the midbody arms and whilst cuts can occur on both sides of the Flemming body, here a single cut is depicted with the asymmetric resorption of the Flemming body by one daughter cell as a midbody remnant.

During cytokinesis, the ESCRT-III associated AAA-ATPase Spastin is recruited to the midbody through interaction with CHMP1B and IST1 [35, 69, 70]. Here, it disassembles midbody microtubules, coordinating the cytoskeletal and membrane remodeling events necessary for abscission. Satisfyingly, during nuclear envelope reformation, Spastin plays a complementary role in disassembling spindle microtubules (which pass through the reforming nuclear envelope), a necessary step before the nuclear envelope is sealed [37^••^]. Here, the CHMP-like protein IST1 was necessary for recruitment of Spastin and subsequent MT disassembly. Disruption of Spastin results in impaired spindle disassembly and persistence of CHMP4B at the re-forming nuclear envelope, indicating that ESCRT-III function at this membrane cannot be completed until MTs penetrating the reassembling nuclear envelope are removed. These results suggest that ESCRT-III and Spastin work together to coordinate nuclear envelope sealing with disassembly of MTs during mitotic exit and highlight a conservation of the machineries that regulate mitotic membrane remodeling events (Figure 4).

These recent findings reveal a new localization and function for ESCRT-III in nuclear envelope remodeling at sites of annular fusion. ESCRT-III may play additional roles at the nuclear envelope — for example, viruses and vesicles containing megaRNPs must transverse this membrane [15, 71, 72] and whilst current models propose alternate transit mechanisms (for example, involving the inter-membrane AAA-ATPase Torsin [73], or viral proteins themselves [74]), ESCRT-III may contribute to this transit. Indeed, it is possible that the surveillance role of ESCRT-III at the yeast nuclear envelope may involve coupling of defective nucleoporin extraction with ESCRT-III-dependent membrane repair. Budding profiles and intra-NE vesicles accumulated in both δ*vps4* yeast and temperature sensitive *npl4* yeast (a UFD1-interacting co-factor of p97 that has been previously implicated in nuclear envelope sealing) [75], indicating parallels may exist between these two functions of ESCRT-III on the nuclear envelope. These data, together with recently described activities of ESCRT-III in the repair of plasma membrane wounds [22•, 23•] points to a role of the ESCRT-III complex in regulating membrane quality — in this case, that of the nuclear envelope. The ESCRT-machinery thus has an important role in the dynamic maintenance of compartmental identity, organelle biogenesis and acts to safeguard the genome from damaging agents. This may be particularly relevant in the context of diseases like laminopathies or cancer, where transient nuclear envelope rupturing and the generation of micronuclei has been reported and is thought to contribute to the disease pathology and development [76, 77, 78, 79].

## Future perspectives

These are exciting times for ESCRT-biology — originally thought to be a key player in the biogenesis of MVBs, the repertoire of physiological and pathophysiological events that involve ESCRT proteins has expanded greatly. In many ways, this reflects the cell's exquisite ability to conserve and repurpose its machineries to accomplish a variety of functions — in this case, those functions that need a topologically equivalent membrane remodeling. ESCRT-III and VPS4 components are conserved as far-back as *Archaea* (prokaryotic organisms lacking membrane bound organelles and nuclei), suggesting that cell division may be considered an ancestral role for the ESCRT-III machinery that evolved as a mechanism to allow binary fission and has been co-opted throughout evolution to allow acquisition of increasingly complex cellular functions [80]. Still, there are many unknowns; whether a surveillance role for ESCRT-III on the nuclear envelope exists in cells performing open mitosis is currently unknown and how a surveillance role for ESCRT-III is coordinated with ESCRT-III-dependent sealing of the reforming nuclear envelope remains an area of open investigation. ESCRT-III is also a key regulator of an Aurora B-dependent cytokinesis checkpoint that is engaged to delay abscission when mitotic defects (including lagging chromosomes [52, 81] and defective NPC assembly [82, 83, 84]) are detected and it is possible that a surveillance role for ESCRT-III-dependent extraction of defective nucleoporins is coupled to an inhibition of ESCRT-III-dependent abscission. This abscission checkpoint is Aurora B-dependent, and Aurora B activity has recently been shown to delay closure of holes in the nuclear envelope to allow lagging chromosomes to be incorporated into forming daughter nuclei [85]. Given the role Aurora B plays in regulating ESCRT-III activity in the context of abscission, it is tempting to speculate that a similar regulation of ESCRT-III may occur during this aspect of nuclear envelope reassembly. Mitotic biology is proving a fruitful area in which to explore ESCRT-function and it is likely that mechanistic insight gleaned from these studies will aid our understanding of this important machinery in non-dividing and pathophysiological states.

## References and recommended reading

Papers of particular interest, published within the period of review, have been highlighted as:

• of special interest
•• of outstanding interest

## Figures and Tables

**Table 1:** ESCRT subunits in yeast and mammals Tabular list of ESCRT components in yeast and mammalian cells; in mammalian cells, ESCRT-III components are referred to as CHMPs, due to their identification as *Ch*arged *M*ultivesicular Body *P*roteins [32]/*Ch*romatin *M*odifying *P*roteins (CHMPs) [33]

Complex	Yeast	Mammals
ESCRT-I	Vps23	TSG101
	Vps28	VPS28
	Vps37	VPS37A, VPS37B, VPS37C, VPS37D
	Mvb12	MVB12A, MVB12B, UBAP1

ESCRT-II	Vps22	EAP30
	Vps25	EAP20
	Vps36	EAP45

ESCRT-III	Did2/Vps46	CHMP1A, CHMP1B
	Vps2	CHMP2A, CHMP2B
	Vps24	CHMP3
	Snf7	CHMP4A, CHMP4B, CHMP4C
	Vps60	CHMP5
	Vps20	CHMP6
	Chm7	CHMP7

ESCRT associated	Vps4	VPS4A, VPS4B
	Vta1	LIP5
	Bro1	ALIX, HD-PTP
	Ist1	IST1
	?	SPASTIN
